# Bridging Environmental Impact and Patient Outcomes: Comment on "A Review of the Applicability of Current Green Practices in Healthcare Facilities"

**DOI:** 10.34172/ijhpm.9138

**Published:** 2025-08-19

**Authors:** Connie Cai Ru Gan, Hansoo Kim

**Affiliations:** ^1^Griffith Institute for Human and Environmental Resilience (GIHER), Griffith University, Brisbane, QLD, Australia.; ^2^School of Medicine and Dentistry, Griffith University, Gold Coast, QLD, Australia.

**Keywords:** Climate Change, Sustainable Healthcare, Planetary Health, Circular Economy, Environmental Impact, Healthcare Facilities

## Abstract

The intersection of healthcare sustainability and clinical practice presents complex challenges in implementing circular economy (CE) principles. This commentary examines Soares and colleagues review of green practices in healthcare facilities while identifying significant gaps in the current discourse. While healthcare facilities are adopting sustainability measures like renewable energy and efficiency improvements, the implementation faces significant operational challenges, particularly in embedding environmental considerations in the clinical decision-making and care delivery process. The analysis discusses that overdiagnosis and low-value care contribute substantially to healthcare’s environmental footprint, exemplified through screening cases that demonstrate the delicate balance between clinical necessity and resource utilisation. We emphasize the need for context-specific approaches that acknowledge operational realities and stakeholder diversity within healthcare governance. We advocate for an integrated approach that places health outcomes at the center of climate initiatives, recognising that public health interventions must equally consider environmental impacts. Ultimately, we call for a paradigm shift that moves beyond siloed environmental initiatives toward systemic integration that complements rather than competes with clinical responsibilities.

 The Healthy Hospital Movement, which began in the late 20th century, laid the foundation for sustainable healthcare practices. This initiative evolved significantly when in 2021, the World Health Organization (WHO) revised its definition of Health Promoting Hospitals and Health Services to incorporate the phrase “support sustainable societies.”^1^ This change reflects a critical recognition of healthcare’s dual role as both affecting and being affected by climate change, emphasising the need for sustainable approaches in healthcare delivery. Over 70% of healthcare carbon footprint comes from indirect sources which are generated through healthcare delivery including supply chains and clinical practices.^2^ This presents healthcare institutions with a significant opportunity to implement circular economy (CE) principles through waste reduction strategies, resource optomisation methodologies, and the development of closed-loop supply chains systems. However, there remains a notable gap in embedding environmental considerations and CE practices in clinical decision-making. This includes how prevention measures, treatment choices, and recovery practice can be optimised for patient outcomes while promoting environmental sustainability through circular design principles.

 In their review paper titled “*A Review of the Applicability of Current Green Practices in Healthcare Facilities*,” Soares et al^3^ present a comprehensive analysis of the CE framework and its potential applications within clinical environments. The authors systematically identify several critical domains for environmental impact reduction in healthcare facilities: Waste management protocols, energy utilisation strategies, water conservation measures, transportation systems, architectural design principles, food sourcing practices, procurement policies, and staff behavioural interventions. The applicability of these environmental initiatives is largely dependent upon green policies and active engagement of healthcare personnel in environmental stewardship practices. The review’s coverage of care delivery related green practices were focusses on telehealth use and patient travel changes. It underrepresents research on environmental performances for specific clinical procedures, likely due to limited studies on facility-level implementation and monitoring. Though the authors analyse practices through CE principles, their findings highlight opportunities for improved systematic tracking and reporting mechanisms at the facility level. It provides limited discussion of service provision and care quality adaptations, especially regarding the applicability of green practices during extreme weather events or health emergencies.

 While these siloed approaches often focus on specific areas of emission sources, literature suggests that significant reductions in healthcare-related emissions could be achieved through evaluation and mitigation of overdiagnosis and low-value care.^4-6^ Unnecessary medical interventions not only create avoidable environmental impacts but also potentially expose patients to risks without proportionate clinical benefits. The challenge, however, is not with the medical interventions themselves, but rather with the structural limitations and incentive systems that drive clinical decision-making. This tension raises a fundamental question about healthcare delivery in an era of climate consciousness: How can we balance our environmental responsibility with our ethical obligation to provide life-saving treatments?

 Beltrán et al^7^ examines overdiagnosis in prostate cancer screening, particularly among older men with elevated prostate-specific antigen levels and significant comorbidities. It emphasises the importance of considering patient comorbidities and overdiagnosis risks before proceeding with prostate-specific antigen testing. In contrast, a systematic review demonstrates the benefits of breast cancer screening, particularly for women aged 50 and older, shows significant reductions in breast cancer mortality through early detection and timely treatment.^8^ While reducing unnecessary medical interventions can decrease healthcare’s environmental footprint, these decisions must be carefully balanced against patient outcomes. The varying evidence regarding intervention effectiveness reflects the nuanced complexity of clinical decision-making. Medical interventions benefit from careful, contextual evaluation that balances immediate patient needs with broader systemic considerations.

 Contemporary research underscores the critical importance of incorporating sustainability principles into healthcare systems through robust ethical frameworks. A study by Moldovan et al^9^ introduced the San-Q framework, which encompasses six domains featuring dual metrics for implementation completion and significance, subsequently validated through hospital-based trials. Rajagopalan et al^10^ conceptualise sustainability as an integral component of organisational strategy, emphasising both environmental impact assessment and institutional transformation towards enhanced equity and inclusivity. The Sustainable Healthcare Coalition^11^ proposed for the systematic integration of environmental considerations within health technology assessments and clinical decision-making processes through standardised methodological approaches. Asperti et al^12^ present “Seventy-Two Shades of Environmental Sustainability,” which represents a paradigmatic shift towards multifaceted sustainability models within healthcare contexts. These theoretical frameworks demonstrate consensus regarding the necessity for comprehensive, ethically-grounded sustainability approaches in healthcare, with particular emphasis on the integration of environmental metrics into strategic planning and governance structures.

 A critical gap in the current discourse lies in addressing the complex operational realities of implementing CE practices and engaging stakeholders effectively. While the review emphasises sustainability teams and staff training, there remain room to further address the inherent tensions between environmental initiatives and the demanding, time-sensitive nature of healthcare delivery. The healthcare governance landscape comprises multiple stakeholders with diverse objectives—from public health providers to private practitioners—each operating under different incentives and constraints. The challenge extends beyond simple implementation to fundamental questions of systemic integration and operational feasibility.

 Furthermore, the assumption that healthcare leaders can seamlessly integrate sustainability initiatives while maintaining core objectives warrants scrutiny. The complexities of implementing CE principles across facility boundaries present significant challenges, particularly in resource-constrained settings.^13^ The current approach to sustainability integration often overlooks the delicate balance required between environmental objectives and clinical imperatives. Healthcare facilities operate under intense pressure to maintain consistent, high-quality care delivery, making any additional procedural requirements potentially disruptive. CE practices must be carefully designed to complement rather than compete with clinical responsibilities – a nuanced consideration often absent from current implementation frameworks. The disconnect between context-specific health priorities and climate-health risk vulnerability creates challenges in balancing resource allocation with patient care quality.

 The proposed competencies in CE impact assessment may not adequately address the inherent tensions between environmental sustainability and immediate healthcare needs. Rather than simply advocating for emission-based environmental benefit, alternative metrics context-relevant approach should consider the ethical implications of communicating environmental metrics over healthcare accessibility. Additionally, while stakeholder engagement is crucial, current training frameworks often underestimate the practical challenges of aligning diverse stakeholder interests, particularly when environmental goals conflict with established clinical practices or resource limitations.

 Lyng et al^14^ analysis of adaptation patterns provides valuable insight into this challenge, distinguishing between reactive “firefighting” approaches and more substantial system reorganisation. Their framework reveals how short-term adaptations, while immediately appealing, often result in superficial changes that may create unintended complications. In contrast, long-term systemic reorganisation, though more challenging to implement, offers the potential for meaningful transformation through feedback-driven improvement. This suggests a necessary evolution in healthcare development: Progressing from tactical adjustments through strategic reorganisation to ultimately achieve genuine innovation in sustainable healthcare delivery.

 Research should examine how CE informed green practices’ affect on patient outcomes—including satisfaction metrics, treatment efficacy, and overall health outcomes—to identify potential trade-offs between sustainability and care quality. Implementation strategies should focus on developing protocols, establishing clear monitoring frameworks, and creating actionable guidelines for healthcare providers to integrate sustainable practices while maintaining high-quality care standards. Financial incentives particularly influence healthcare decisions, as demonstrated by how subsidised medications can effectively guide patient choices toward more sustainable options.

 To facilitate better decision-making, there is a critical need to develop comprehensive evaluation systems that can assess climate, health, and economic impacts of healthcare programs. While Soares and colleagues’^3^ paper establishes a valuable foundation for understanding CE practices’ applicability in healthcare, further research is essential to explore their broader implications for service demand and care quality. Critical areas for future investigation include economic evaluation of CE practice implementation in healthcare settings, encompassing staff training costs, sustainable procurement strategies, and energy-efficient system adoption as decision-makers view this as critical evidence to justify investment in CE implementation. Similar to how infrastructure projects undergo cost-benefit analyses and health interventions are evaluated through cost-utility analyses, healthcare sustainability initiatives require systematic evaluation frameworks. This would enable decision-makers to effectively compare and prioritise different programs while balancing environmental impact with healthcare outcomes.

 Additionally, stakeholder engagement in sustainability initiative development should incorporate these multifaceted evaluation frameworks. Policy recommendations should focus on creating regulatory structures that promote environmentally conscious practices while ensuring healthcare quality and accessibility. Training for healthcare leaders should emphasis this integrated approach, combining sustainability principles with health economics and outcome metrics to optimise both environmental and clinical objectives. While the review paper presents a comprehensive analysis of CE practices’ environmental benefits in healthcare settings, we argued that it needs addressing these systemic issues requires a paradigm shift in the implications for service delivery and care quality warrant further scrutiny. Given that healthcare facilities’ primary mandate is to ensure continuous, high-quality patient care, sustainability initiatives must be carefully evaluated against this fundamental objective.

## Ethical issues

 Not applicable.

## Conflicts of interest

 Authors declare that they have no conflicts of interest.
